# Impact of a Genetic Risk Score for Coronary Artery Disease on Reducing Cardiovascular Risk: A Pilot Randomized Controlled Study

**DOI:** 10.3389/fcvm.2017.00053

**Published:** 2017-08-14

**Authors:** Joshua W. Knowles, Shirin Zarafshar, Aleksandra Pavlovic, Benjamin A. Goldstein, Sandra Tsai, Jin Li, Michael V. McConnell, Devin Absher, Euan A. Ashley, Michaela Kiernan, John P. A. Ioannidis, Themistocles L. Assimes

**Affiliations:** ^1^Division of Cardiovascular Medicine, Cardiovascular Institute, Stanford University School of Medicine, Stanford, CA, United States; ^2^Duke Clinical Research Institute, Duke University, Durham, NC, United States; ^3^Division of General Medical Disciplines, Stanford University School of Medicine, Stanford, CA, United States; ^4^HudsonAlpha Institute for Biotechnology, Huntsville, AL, United States; ^5^Department of Genetics, Stanford University School of Medicine, Stanford, CA, United States; ^6^Stanford University School of Medicine, Stanford Prevention Research Center, Stanford, CA, United States; ^7^Department of Health Research and Policy, Stanford University School of Medicine, Stanford, CA, United States; ^8^Department of Statistics, Stanford University School of Humanities and Sciences, Stanford, CA, United States

**Keywords:** genetic risk score, coronary artery disease, LDL-cholesterol, GWAS, cardiovascular risk

## Abstract

**Purpose:**

We tested whether providing a genetic risk score (GRS) for coronary artery disease (CAD) would serve as a motivator to improve adherence to risk-reducing strategies.

**Methods:**

We randomized 94 participants with at least moderate risk of CAD to receive standard-of-care with (*N* = 49) or without (*N* = 45) their GRS at a subsequent 3-month follow-up visit. Our primary outcome was change in low density lipoprotein cholesterol (LDL-C) between the 3- and 6-month follow-up visits (ΔLDL-C). Secondary outcomes included other CAD risk factors, weight loss, diet, physical activity, risk perceptions, and psychological outcomes. In pre-specified analyses, we examined whether there was a greater motivational effect in participants with a higher GRS.

**Results:**

Sixty-five participants completed the protocol including 30 participants in the GRS arm. We found no change in the primary outcome between participants receiving their GRS and standard-of-care participants (ΔLDL-C: −13 vs. −9 mg/dl). Among participants with a higher GRS, we observed modest effects on weight loss and physical activity. All other secondary outcomes were not significantly different, including anxiety and worry.

**Conclusion:**

Adding GRS to standard-of-care did not change lipids, adherence, or psychological outcomes. Potential modest benefits in weight loss and physical activity for participants with high GRS need to be validated in larger trials.

## Introduction

Coronary artery disease (CAD) remains the leading cause of morbidity and mortality worldwide (1). Primary prevention is a goal for providers everywhere and can result in remarkable reductions in event rates when applied optimally. However, motivation to adhere to prescribed medications and lifestyle changes known to reduce the risk of CAD continues to be a major challenge and contributes substantially to suboptimal outcomes. For example, prior studies have shown poor adherence to statins in both primary and secondary prevention populations with discontinuation rates of up to ~40% (2–4). Other studies such the Nurses’ Health study have documented a high population-attributable risk of CAD events related to suboptimal diet, physical activity, diet, and smoking patterns (5).

One of the cornerstones of prevention counseling and therapy is the estimation of risk for future cardiovascular events. Emerging data suggest that a genetic risk score (GRS) modestly improves risk prediction beyond traditional risk factors included in widely used clinical risk scores such as the Framingham or Omnibus risk estimator (6–9). The GRS summarizes the degree of exposure to high-risk alleles for CAD identified through genome-wide association studies. The clinical utility of these observations remains unclear in the absence of randomized trials that directly document a clinical benefit (e.g., reduction in CAD events through the incorporation of a GRS) given the degree of incremental improvement of risk prediction provided by the GRS remains quite modest (6–9).

Enhanced risk prediction may ultimately serve as a primary means by which a GRS improves outcomes, but the communication of one’s genetic risk may also improve outcomes by motivating participants to better adhere to proven primary prevention strategies. This hypothesis has been tested for CAD using other novel biomarkers such as coronary artery calcification (CAC) with some recent evidence of benefit (10). On the other hand, providing participants with their genetic risk in other settings such as type 2 diabetes was not found to be more effective than conventional counseling (11). A recent trial demonstrated a modest effect on the use of GRS in influencing shared decision-making resulting in more statin prescriptions (12). However, the trial design did not allow a differentiation between the effect on the health-care provider or the patient. To provide more evidence in this regard, we conducted a pilot randomized clinical trial in participants at risk of CAD to ascertain the utility of a GRS as a motivational tool to reduce risk factors for CAD in a routine clinical setting over a 3-month period.

## Patients and Methods

### Population and Study Design

We have previously published the details of the study design and protocol (13). The protocol was registered at https://ClinicalTrials.gov NCT01406808 and was approved by the Stanford Institutional Review Board, and all participants gave informed consent. We recruited participants from the Stanford Preventive Cardiology Clinic, and the trial was advertised through the disbursement of flyers to local cardiologists, general practitioners, and family medicine doctors. Enrollment began in August 2011 and follow-up was completed in April 2015.

Participants seeking cardiovascular risk evaluation or optimization either through self-referral or referral from another provider who had at least a 6% risk of CAD over the next 10 years or >20% risk over the next 30 years as estimated by the Framingham risk score were eligible to participate (13). Participants also had to be White, South-Asian, or Hispanic/Latino to be eligible to participate as these race/ethnic groups have similar genetic architecture for the risk alleles that were used (13–15). Exclusion criteria included a history of atherosclerotic myocardial infarction, angina, bypass surgery, percutaneous intervention, stroke, peripheral arterial disease, active statin therapy, previous genetic testing, medical conditions that would limit ability to adhere to recommendations, and <1 year anticipated survival (13).

Eligible participants were randomized to receive either standard-of-care or standard-of-care with the addition of GRS delivered by the physician using a suggested script. Participants were randomized using a permuted block algorithm with a block size of 8. The risk assessment at baseline that did not consider an individual’s genetic risk dictated the primary prevention recommendations for all participants. We used the National Cholesterol Education Program/Adult Treatment Panel III guidelines to determine the participant’s goal low density lipoprotein cholesterol (LDL-C) and medications as needed. The trial was designed and recruitment was mostly completed before the publication of the 2013 AHA/ACC lipid guidelines (16).

We genotyped all participants with Illumina’s iSelect Cardio-Metabochip array between the baseline visit and their second visit scheduled approximately 3 months after their baseline visit at the HudsonAlpha Institute of Biotechnology Genomic Services Laboratory (13). We used a total of 19 SNPs on the array associated with the risk of CAD independent of traditional risk factors to construct a weighted GRS for all participants and used the GRS to update the 10-year Framingham risk of all participants (13, 17). Briefly, we first derived a normalized score of the number of high-risk alleles possessed by each white participant in the ARIC study (*n* = 8,734) using the respective log odds ratios for each of the 19 SNPs from CARDIoGRAM as weights (13). This distribution of scores served as the population-based comparison for the participant’s GRS. To assess the 10-year risk of CVD based on an individual’s GRS, a relative risk regression was estimated within the ARIC cohort, adjusting for sex and age. Within the ARIC sample, the relative risk for a 1 SD increase in genetic risk was found to be 1.18 (95% confidence intervals: 1.12, 1.25). For participants in this study, a GRS was calculated in the same manner as it was calculated for ARIC cohort members (13). Each individual’s 10-year risk of CHD based on the Framingham risk calculator was computed. This 10-year risk was then multiplied by the individual’s estimated genetic relative risk to generate an updated risk. The 19 SNPs included in the GRS were as follows: rs17465637, rs9970807 (proxy for rs17114036), rs6725887, rs2306374, rs12190287, rs12204265 (proxy for rs17609940), rs12526453, rs11556924, rs4977574, rs1746048, rs2246833, rs2505083, rs974819, rs4773144, rs2895811, rs7177699 (proxy for rs3825807), rs12449964 (proxy for rs12936587), rs143499 (proxy for rs216172), and rs9305545 (proxy for rs9982601) (13).

We gave providers (previously blinded to the results of the GRS) a sealed envelope containing either the results of genetic testing for participants randomized to the GRS arm or a simple statement reminding participants in the standard-of-care arm that they will only receive their results after they complete the study. Providers explained the GRS and updated risk using a script and a standardized figure (13, 17). Primary prevention recommendations set at the baseline visit were not altered by the results of the genetic testing. Finally, we asked all participants to undergo lipid testing at the Stanford lab after their second clinic visit.

We scheduled a third and final study visit approximately 6 months after the baseline visit. Participants randomized to the standard-of-care arm received the results of their genetic testing at the conclusion of the third visit.

### Outcomes

The primary outcome was the change in LDL-C between visits 2 and 3 between the standard-of-care and the GRS arm. Secondary outcomes included changes in all other lipid measures as well, other changes in other traditional risk factors including weight, blood pressure (BP), leisure-time physical activity, and dietary intake. We also assessed medication adherence, attitudes toward taking prescribed medicines, anxiety, and stages of change.

### Statistical Analysis

We used standard methods to calculate summary statistics for characteristics at baseline as well all outcomes at the 3-month visit and the 6-month visit stratified by randomization arm. Next, we calculated standard summary statistics for the distribution of differences in repeated measures between the 3- and 6-month visits stratified by randomization arm. Finally, we calculated Hodges–Lehmann derived medians of these differences within each arm and tested whether population medians were different from each other with a Hodges–Lehmann statistic.

Incomplete responses to questionnaires were not uncommon as was a failure for participants to undergo lipid testing. To maximize power and minimize drop out from our analyses, we carried forward partial responses from questionnaires completed during the baseline visit to the second and/or third visit when they were not available assuming that the response for that question did not change from the prior visit(s). When a questionnaire was missing in its entirety at the third visit as well as either the first or second visit, the individual was excluded from analyses for that questionnaire. For lipids, we carried forward results from the baseline visit when results from visit 2 were not available. Participants who did not have a lipid panel test soon after the final visit were excluded from analyses.

We conducted two pre-specified subgroup analyses (13). The first involved the subgroup of participants in the GRS arm with a GRS above the population median leading to an increased updated 10-year Framingham risk. The second subgroup involved the subset participants in either arm with positive attitudes toward taking medications. The median of aggregate of the Likert scores from the beliefs about medicines questionnaire was used to identify this subgroup.

## Results

**Figure 1:**
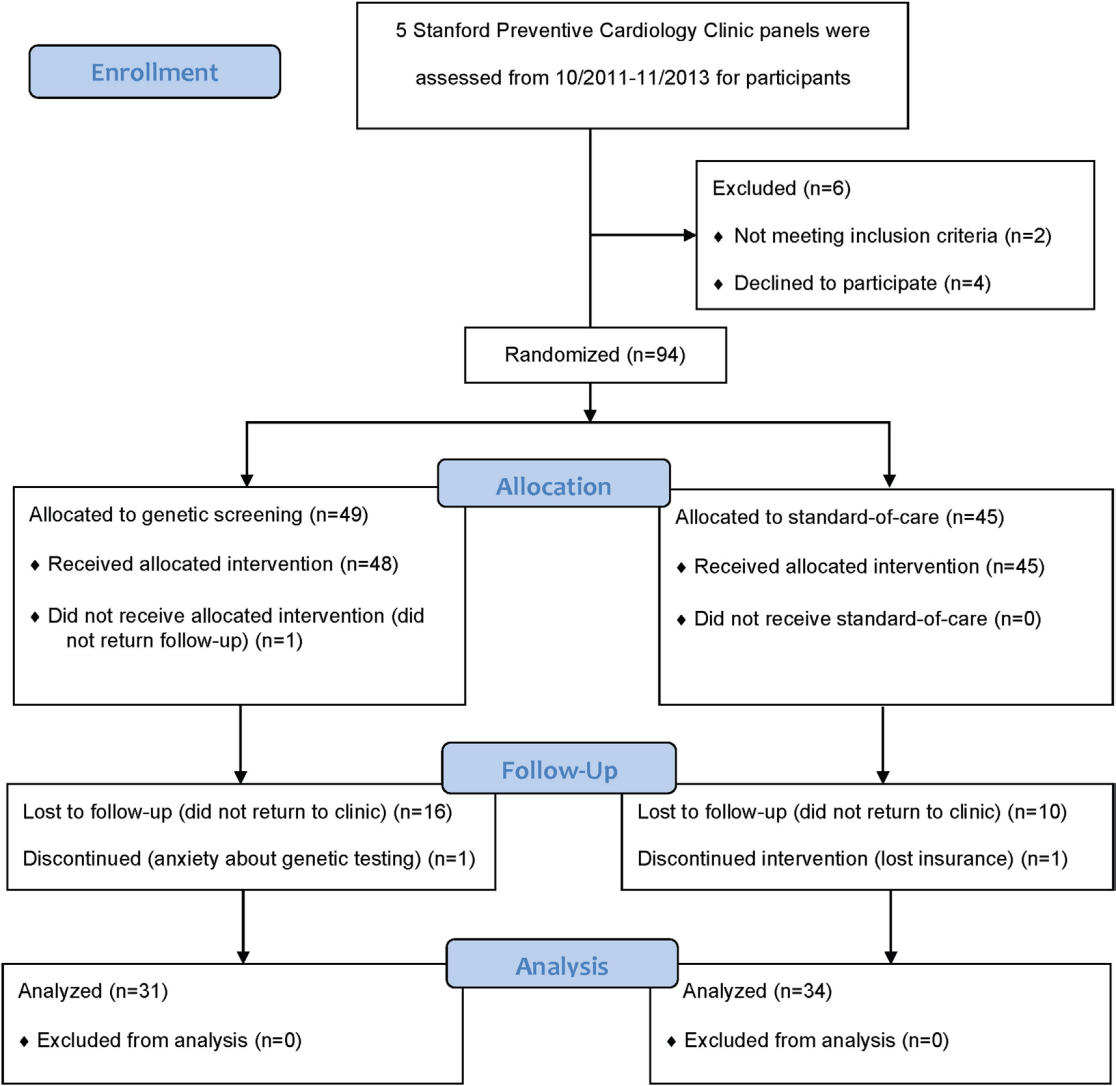
CONSORT flow diagram.

Providers consented a total of 100 participants between October 2011 and November 2013. We subsequently withdrew two participants because they did not meet all eligibility criteria. An additional four consented participants withdrew prior to submitting their biospecimen at the baseline visit (Figure 1). Of the remaining 94 participants, we randomized 49 to the GRS arm and 45 to the standard-of-care arm. We summarize the distribution of baseline characteristics in Table 1. A total of 77 participants (82%) attended the 3-month follow-up visit and 65 participants (69%) attended the 6-month final follow-up visit. A total of 17 participants in the GRS arm and 11 in the standard-of-care arm did not return to clinic to complete the study (*p* = 0.14). In the GRS arm, the mean, median, SD, minimum, and maximum number of high-risk alleles were 18.1, 18, 2.5, 12, and 24, respectively. Among the participants in standard-of-care arm, these numbers were similar at 18.4, 18, 2.4, 11, and 24.

**Table 1:** Baseline demographics of randomized participants.

Characteristic	GRS (*n* = 49)	Standard-of-care (*n* = 45)
Age, years (SD)	57 (±10)	58 (±8)
Female (%)	39	47
Weight, kg (SD)	85 (±17)	86 (±21)
Race/ethnicity (%)		
White	73	78
South-Asian	12	2
Hispanic/Latino	6	13
Middle Eastern	8	2
Other	0	4
10-year Framingham risk (%)	11 (±8)	12 (±7)
Systolic BP (mmHg)	124 (±15)	134 (±21)
Diastolic BP (mmHg)	78 (±9)	80 (±11)
Diabetes (%)	8	4
Total cholesterol (mg/dl)	220 (±40)	220 (±45)
LDL-C (mg/dl)	141 (±37)	138 (±39)
HDL-C (mg/dl)	50 (±18)	57 (±21)
Triglycerides (mg/dl)	164 (±133)	134 (±77)
Anti-HTN use (%)^a^	26	36
Non-statin lipid therapy (%)^b^	15	17

*LDL-C, low density lipoprotein cholesterol; HDL-C, high density lipoprotein concentration; BP, blood pressure; GRS, genetic risk score*.

*^a^Data available in 38 participants in GRS and 42 in standard-of-care arm*.

*^b^Data available in 40 participants in GRS and 42 in standard-of-care arm*.

Table 2 summarizes the results of our primary and secondary outcomes. We observed no significant difference in the degree of LDL-C reduction between the standard-of-care arm and the GRS arm participants with available lipid data. We found no significant differences in the degree of changes in high density lipoprotein concentration, BP, weight, physical activity, diet, anxiety over genetic testing, or stages of change between the two groups. With respect to medication use, we found no significant difference in the proportion of participants on statins at the time of the 3-month visit (20% in the GRS vs. 26% in the standard-of-care arm, *p* = 0.59) as well as the 6-month visit (23% in the GRS vs. 20% in the standard-of-care arm, *p* = 0.75). Similarly, we found no difference in the proportion of participants on antihypertensive drugs at the time of the 3-month visit (23% in the GRS vs. 40% in the standard-of-care arm, *p* = 0.15) as well as the 6-month visit (27% in the GRS vs. 34% in the standard-of-care arm, *p* = 0.51).

**Table 2:** Primary and secondary outcomes among GRS and standard-of-care arms.

	GRS				Standard-of-care					
	*n*	3-month visit mean ± SD	6-month visit mean ± SD	Δ 3- to 6-month mean ± SD	*n*	3-month visit mean ± SD	6-month visit mean ± SD	Δ 3- to 6-month mean ± SD	Difference (95% CI) in median between groups	*p* for Difference in median ≠ 0
**Primary outcome**										
LDL-C (mg/dl), imputation	27	132 ± 23	118 ± 33	−13 ± 29	34	125 ± 42	116 ± 40	−9 ± 27	−5.5 (−20, 10)	0.86
LDL-C (mg/dl), no imputation	22	132 ± 24	125 ± 32	−7 ± 27	27	124 ± 44	114 ± 40	−10 ± 28	2.5 (−14, 19)	0.46
**Secondary outcomes**										
HDL-C (mg/dl)	27	54 ± 19	55 ± 18	1 ± 9	34	55 ± 14	54 ± 14	−1 ± 9	1.5 (−4, 7)	0.66
Systolic BP (mmHg)	30	128 ± 16	128 ± 15	0.8 ± 13	35	132 ± 20	130 ± 21	−2 ± 14	4.5 (−2, 11)	0.18
Diastolic BP (mmHg)	30	77 ± 9	77 ± 10	0.1 ± 11	35	79 ± 10	79 ± 11	0.3 ± 10	0 (−5, 5)	0.91
Weight (kg)	30	83 ± 20	82 ± 19	−1 ± 4	35	84 ± 16	84 ± 17	−0.1 ± 3	−0.8 (−1.9, 0.3)	0.21
Diet^a^	21	3.4 + 0.6	3.3 + 0.7	0.3	28	3.3 + 0.7	3.3 + 0.7	−0.03 + 0.8	−0.10 (−0.46, 0.27)	0.44
Physical activity^b^	23	3.4 + 1.6	3.7 + 1.4	0.3 + 1.2	29	3.9 + 1.3	3.8 + 1.3	−0.2 + 0.8	0.5 (−1.1)	0.20
Anxiety over genetic testing	24	1.7 ± 0.3	1.7 ± 0.7	−0.03 ± 0.3	29	1.7 ± 0.4	1.7 ± 0.5	−0.04 ± 0.6	−0.12 (−0.3, 0.17)	0.47
Stages of change	25	4.1 ± 0.8	4.0 ± 0.7	0.1 ± 0.7	30	4.0 ± 1.0	3.7 ± 0.8	−0.4 ± 0.9	0.25 (0.0, 0.68)	0.10

*LDL-C, low density lipoprotein cholesterol; HDL-C, high density lipoprotein concentration; BP, blood pressure; GRS, genetic risk score*.

*^a^Scored from 0 to 5 with improved diet associated with higher scores*.

*^b^Six categories of increasing physical activity, coded 1–6*.

Table 3 summarizes the results of our pre-specified subgroup analyses. For the subgroup of high genetic risk, we found no significant differences in the primary outcome of LDL-C but we observed modest beneficial effects for weight loss and physical activity among participants in the GRS arm compared to the standard-of-care arm. Among participants with a positive attitude toward medications, we did not observe any significant differences in the change in LDL-C between the two arms.

**Table 3:** Subgroup analysis of primary and select secondary outcomes restricted to either high GRS (>50th percentile) or positive attitude toward taking medications (>50th percentile).

	GRS				Standard-of-care					
	*n*	3-month visit mean ± SD	6-month visit mean ± SD	Δ 3- to 6-month mean ± SD	*n*	3-month visit mean ± SD	6-month visit mean ± SD	Δ 3- to 6-month mean ± SD	Difference (95% CI) in median between groups	*p* for Difference in median ≠ 0
High genetic risk										
LDL-C (mg/dl)	13	126 ± 24	109 ± 34	−17 ± 31	34	125 ± 42	116 ± 40	−9 ± 28	−10.5 (−29, 12)	0.37
Weight (kg)	12	79 ± 19	77 ± 18	−2.3 ± 3	34	83 ± 16	83 ± 16	0.0 ± 3	−1.9 (−3.9, −0.4)	0.02
Physical activity^a^	8	2.6 ± 0.5	3.3 ± 1.0	0.6 ± 1.1	29	3.9 ± 1.3	3.8 ± 1.3	−0.2 ± 0.8	0.5 (0, 2)	0.04
Positive attitude toward meds										
LDL-C (mg/dl)	11	135 ± 22	127 ± 31	−8 ± 25	16	121 ± 44	118 ± 40	−3 ± 24	−7.5 (−26, 16)	0.40

*LDL-C, low density lipoprotein cholesterol; GRS, genetic risk score*.

*^a^Six categories of increasing physical activity, coded 1–6*.

## Discussion

We examined whether communicating DNA-based risk estimates of CAD through a brief structured encounter not involving a genetic counselor would have a beneficial effect on health-related behavior leading to an improved cardiovascular risk profile beyond that resulting from a standard-of-care risk assessment. We found no significant difference between the two arms for our primary and secondary outcomes. Among the subgroup with higher genetic risk, we found modest beneficial effects on weight loss and physical activity, but these latter findings should be interpreted with caution given the number of statistical comparisons performed. Finally, we demonstrated that genetic information for a complex polygenic trait like CAD can be provided in a busy clinical practice with no significant adverse psychological effects.

Our findings are consistent with a recently published meta-analysis of 18 randomized and quasi-randomized controlled trials involving adults receiving genetic based estimates of risk for conditions whose risk can be influenced by a change in behavior (18). Researchers concluded that genetic data does not result in large motivating or demotivating changes in risk-reducing behavior but they could not rule out smaller effects given the limited number and size of trials reported to date. Our findings are also consistent with a cohort study reporting no impact on physical activity, diet, or psychological health as a consequence of direct-to-consumer genome-wide genetic profiling that was used to estimate the lifetime risk of 22 medical conditions (19).

A recently completed randomized control trial (MI-GENES) of 203 participants with similar inclusion criterion to our study found that disclosure of 10-year risk estimates incorporating a GRS of CAD resulted in LDL-C levels that were ~10 mg/dl lower among participants with high genetic risk compared to participants undergoing risk assessment restricted to conventional risk factors (12). A key distinction between the design of the MI-GENES study and our study was how risk estimates incorporating an individual’s genetic risk were used in the decision to prescribe statins. In MI-GENES, these risk estimates were used to guide the recommendations for statins and were likely largely responsible for the higher rate of statin prescriptions and the lower LDL-C levels in the high genetic risk group. In contrast, providers in our study were blinded to the results of the GRS until after recommendations for statin therapy had been established using risk estimates that did include genetic risk. Our approach allowed us to separate out any beneficial effect of the GRS on LDL-C levels related to increased adherence to risk-reducing strategies from the effect related to an increase rate of statin prescriptions as a consequence of elevated 10-year risk.

We note that initial smaller studies using CAC scores and images to motivate adherence were also unable to detect a beneficial effect over usual care but more recent studies, including a large randomized clinical trial, have demonstrated clinically meaningful benefits in adherence to medications and reduction in cardiovascular risk (20–22). Thus, larger studies are necessary to confirm or refute our findings. Of interest, a recent observational study suggests that adherence to an optimal lifestyle that includes a combination of physical activity at least once a week, maintaining a BMI < 30, no smoking, and a healthy diet reduces one’s risk of CAD irrespective of one’s baseline genetic risk. Furthermore, the effects of an optimal lifestyle are able to easily overcome even the most excess genetic risk (23). Future studies may be more effective in motivating behavioral change as the GRS is expected to improve its predictive ability through the incorporation of novel CAD loci.

Our study had several limitations. First, the 52% retention rate was less than expected which lowered our power to detect a difference of the degree of LDL-C lowering between the two arms as well as the change in other secondary outcomes (13). Multiple factors may have contributed including participant movement, loss-of-insurance, and our inability to fund lipid testing. Second, although we assessed physical activity with a single categorical item previously shown to be sensitive to change among inactive individuals (24) and similarly shown here to be sensitive to change among the subgroup of participants with high GRSs, self-reported assessments of health behaviors in general can be subject to recall bias. Finally, we assessed the effects of our intervention over a limited 3-month period. A longer follow-up period would have provided us with the opportunity to assess whether effects of the GRS on physical activity and weight gain observed in the first 3 months would have faded or would have been sustained and contributed to an improved LDL-C over time independent of statin therapy.

In conclusion, we observed no major effect of communicating genetic risk for CAD on risk-reducing health behaviors. The potential benefits in weight loss and physical activity for participants with high GRSs need to be validated in larger and longer term trials. The clinical utility of a GRS of CAD remains unclear in the absence of large-scale clinical trials demonstrating a clear benefit in adherence or in the reduction of CAD events among participants whose overall risk and treatment recommendations has been directly informed by their GRS.

## Author Contributions

JK: conceived of the original design, had day-to-day operational control of the trial as well as performed primary writing and editing of the manuscript. SZ: contributed to data analysis, writing and editing of the manuscript. AP: oversaw recruitment for the trial, data management, and analysis. BG: assisted with data analysis and trial design. ST: contributed to study design and recruitment. JL: assisted with data analysis. MM: contributed to the design and oversight of the trial and assisted with revisions to the manuscript. DA: oversaw genotyping in the trial and contributed to design of the trial. EA: helped to conceive of the trial, assisted with trial design, data analysis, and writing. MK: contributed to the design of the trial, particularly the design of the questionnaires as well as provided assistance with data analysis and writing of the manuscript. JI: helped to conceive of the trial, assisted with trial design, data analysis, and writing and editing the manuscript. TA: helped to conceive of the trial, assisted with trial design, oversaw primary data analysis, and writing and editing the manuscript.

## Conflict of Interest Statement

MM is now employed at Verily, Inc. All other authors declare that the research was conducted in the absence of any commercial or financial relationships that could be construed as a potential conflict of interest.

## References

[1] Mozaffarian D Benjamin EJ Go AS Arnett DK Blaha MJ Cushman M Heart disease and stroke statistics – 2015 update: a report from the American Heart Association. Circulation (2015) 131(4):e29–322. 10.1161/CIR.0000000000000152 25520374

[2] Ellis JJ Erickson SR Stevenson JG Bernstein SJ Stiles RA Fendrick AM. Suboptimal statin adherence and discontinuation in primary and secondary prevention populations. J Gen Intern Med (2004) 19(6):638–45. 10.1111/j.1525-1497.2004.30516.x 15209602 PMC1492382

[3] Tang L Patao C Chuang J Wong ND. Cardiovascular risk factor control and adherence to recommended lifestyle and medical therapies in persons with coronary heart disease (from the National Health and Nutrition Examination Survey 2007-2010). Am J Cardiol (2013) 112(8):1126–32. 10.1016/j.amjcard.2013.05.064 23827404

[4] Iqbal J Zhang YJ Holmes DR Morice MC Mack MJ Kappetein AP Optimal medical therapy improves clinical outcomes in patients undergoing revascularization with percutaneous coronary intervention or coronary artery bypass grafting: insights from the synergy between percutaneous coronary intervention with TAXUS and cardiac surgery (SYNTAX) trial at the 5-year follow-up. Circulation (2015) 131(14):1269–77. 10.1161/CIRCULATIONAHA.114.013042 25847979

[5] Stampfer MJ Hu FB Manson JE Rimm EB Willett WC. Primary prevention of coronary heart disease in women through diet and lifestyle. N Engl J Med (2000) 343(1):16–22. 10.1056/NEJM200007063430103 10882764

[6] Tikkanen E Havulinna AS Palotie A Salomaa V Ripatti S. Genetic risk prediction and a 2-stage risk screening strategy for coronary heart disease. Arterioscler Thromb Vasc Biol (2013) 33(9):2261–6. 10.1161/ATVBAHA.112.301120 23599444 PMC4210840

[7] Ganna A Magnusson PK Pedersen NL de Faire U Reilly M Arnlöv J Multilocus genetic risk scores for coronary heart disease prediction. Arterioscler Thromb Vasc Biol (2013) 33(9):2267–72. 10.1161/ATVBAHA.113.301218 23685553

[8] Mega JL Stitziel NO Smith JG Chasman DI Caulfield M Devlin JJ Genetic risk, coronary heart disease events, and the clinical benefit of statin therapy: an analysis of primary and secondary prevention trials. Lancet (2015) 385(9984):2264–71. 10.1016/S0140-6736(14)61730-X 25748612 PMC4608367

[9] Tada H Melander O Louie JZ Catanese JJ Rowland CM Devlin JJ Risk prediction by genetic risk scores for coronary heart disease is independent of self-reported family history. Eur Heart J (2016) 37(6):561–7. 10.1093/eurheartj/ehv462 26392438 PMC4744619

[10] Joseph PG Pare G Asma S Engert JC Yusuf S Anand SS Impact of a genetic risk score on myocardial infarction risk across different ethnic populations. Can J Cardiol (2016) 32(12):1440–6. 10.1016/j.cjca.2016.05.014 27650930

[11] Voils CI Coffman CJ Grubber JM Edelman D Sadeghpour A Maciejewski ML Does type 2 diabetes genetic testing and counseling reduce modifiable risk factors? A randomized controlled trial of veterans. J Gen Intern Med (2015) 30(11):1591–8. 10.1007/s11606-015-3315-5 25876740 PMC4617940

[12] Kullo IJ Jouni H Austin EE Brown SA Kruisselbrink TM Isseh IN Incorporating a genetic risk score into coronary heart disease risk estimates: effect on low-density lipoprotein cholesterol levels (the MI-GENES clinical trial). Circulation (2016) 133(12):1181–8. 10.1161/CIRCULATIONAHA.115.020109 26915630 PMC4803581

[13] Knowles JW Assimes TL Kiernan M Pavlovic A Goldstein BA Yank V Randomized trial of personal genomics for preventive cardiology: design and challenges. Circ Cardiovasc Genet (2012) 5(3):368–76. 10.1161/CIRCGENETICS.112.962746 22715281 PMC3394683

[14] Vargas JD Manichaikul A Wang XQ Rich SS Rotter JI Post WS Common genetic variants and subclinical atherosclerosis: the multi-ethnic study of atherosclerosis (MESA). Atherosclerosis (2016) 245:230–6. 10.1016/j.atherosclerosis.2015.11.034 26789557 PMC4738145

[15] Coronary Artery Disease (C4D) Genetics Consortium. A genome-wide association study in Europeans and South Asians identifies five new loci for coronary artery disease. Nat Genet (2011) 43(4):339–44. 10.1038/ng.782 21378988

[16] Stone NJ Robinson JG Lichtenstein AH Bairey Merz CN Blum CB Eckel RH 2013 ACC/AHA guideline on the treatment of blood cholesterol to reduce atherosclerotic cardiovascular risk in adults: a report of the American College of Cardiology/American Heart Association Task Force on practice guidelines. Circulation (2014) 129(25 Suppl 2):S1–45. 10.1161/01.cir.0000437738.63853.7a 24222016

[17] Goldstein BA Knowles JW Salfati E Ioannidis JP Assimes TL. Simple, standardized incorporation of genetic risk into non-genetic risk prediction tools for complex traits: coronary heart disease as an example. Front Genet (2014) 5:254. 10.3389/fgene.2014.00254 25136350 PMC4117937

[18] Hollands GJ French DP Griffin SJ Prevost AT Sutton S King S The impact of communicating genetic risks of disease on risk-reducing health behaviour: systematic review with meta-analysis. BMJ (2016) 352:i1102. 10.1136/bmj.i1102 26979548 PMC4793156

[19] Bloss CS Schork NJ Topol EJ. Effect of direct-to-consumer genomewide profiling to assess disease risk. N Engl J Med (2011) 364(6):524–34. 10.1056/NEJMoa1011893 21226570 PMC3786730

[20] Lederman J Ballard J Njike VY Margolies L Katz DL. Information given to postmenopausal women on coronary computed tomography may influence cardiac risk reduction efforts. J Clin Epidemiol (2007) 60(4):389–96. 10.1016/j.jclinepi.2006.07.010 17346614

[21] Mamudu HM Paul TK Veeranki SP Budoff M. The effects of coronary artery calcium screening on behavioral modification, risk perception, and medication adherence among asymptomatic adults: a systematic review. Atherosclerosis (2014) 236(2):338–50. 10.1016/j.atherosclerosis.2014.07.022 25128971

[22] Kalia NK Cespedes L Youssef G Li D Budoff MJ. Motivational effects of coronary artery calcium scores on statin adherence and weight loss. Coron Artery Dis (2015) 26(3):225–30. 10.1097/MCA.0000000000000207 25514570

[23] Khera AV Emdin CA Drake I Natarajan P Bick AG Cook NR Genetic risk, adherence to a healthy lifestyle, and coronary disease. N Engl J Med (2016) 375(24):2349–58. 10.1056/NEJMoa1605086 27959714 PMC5338864

[24] Kiernan M Schoffman DE Lee K Brown SD Fair JM Perri MG The Stanford leisure-time activity categorical item (L-Cat): a single categorical item sensitive to physical activity changes in overweight/obese women. Int J Obes (Lond) (2013) 37(12):1597–602. 10.1038/ijo.2013.36 23588625 PMC4731089

